# Regional contributions of D-serine to Alzheimer’s disease pathology in male *App*^NL–G–F/NL–G–F^ mice

**DOI:** 10.3389/fnagi.2023.1211067

**Published:** 2023-06-29

**Authors:** Xiance Ni, Ran Inoue, Yi Wu, Tomoyuki Yoshida, Keisuke Yaku, Takashi Nakagawa, Takashi Saito, Takaomi C. Saido, Keizo Takao, Hisashi Mori

**Affiliations:** ^1^Department of Molecular Neuroscience, Faculty of Medicine, University of Toyama, Toyama, Japan; ^2^Graduate School of Innovative Life Science, University of Toyama, Toyama, Japan; ^3^Research Center for Idling Brain Science, University of Toyama, Toyama, Japan; ^4^Department of Molecular and Medical Pharmacology, Faculty of Medicine, University of Toyama, Toyama, Japan; ^5^Research Center for Pre-Disease Science, University of Toyama, Toyama, Japan; ^6^Laboratory for Proteolytic Neuroscience, RIKEN Center for Brain Science, Saitama, Japan; ^7^Department of Neurocognitive Science, Institute of Brain Science, Nagoya City University Graduate School of Medical Sciences, Aichi, Japan; ^8^Department of Behavioral Physiology, Faculty of Medicine, University of Toyama, Toyama, Japan

**Keywords:** Alzheimer’s disease, D-serine, serine racemase, excitotoxicity, neurodegeneration, amino acid homeostasis

## Abstract

**Background:**

Neurodegenerative processes in Alzheimer’s disease (AD) are associated with excitotoxicity mediated by the *N*-methyl-D-aspartate receptor (NMDAR). D-Serine is an endogenous co-agonist necessary for NMDAR-mediated excitotoxicity. In the mammalian brain, it is produced by serine racemase (SRR) from L-serine, suggesting that dysregulation of L-serine, D-serine, or SRR may contribute to AD pathogenesis.

**Objective and methods:**

We examined the contributions of D-serine to AD pathology in the *App^NL–G–F/NL–G–F^* gene knock-in (APPKI) mouse model of AD. We first examined brain SRR expression levels and neuropathology in APPKI mice and then assessed the effects of long-term D-serine supplementation in drinking water on neurodegeneration. To further confirm the involvement of endogenous D-serine in AD progression, we generated *Srr* gene-deleted APPKI (APPKI-SRRKO) mice. Finally, to examine the levels of brain amino acids, we conducted liquid chromatography–tandem mass spectrometry.

**Results:**

Expression of SRR was markedly reduced in the retrosplenial cortex (RSC) of APPKI mice at 12 months of age compared with age-matched wild-type mice. Neuronal density was decreased in the hippocampal CA1 region but not altered significantly in the RSC. D-Serine supplementation exacerbated neuronal loss in the hippocampal CA1 of APPKI mice, while APPKI-SRRKO mice exhibited attenuated astrogliosis and reduced neuronal death in the hippocampal CA1 compared with APPKI mice. Furthermore, APPKI mice demonstrated marked abnormalities in the cortical amino acid levels that were partially reversed in APPKI-SRRKO mice.

**Conclusion:**

These findings suggest that D-serine participates in the regional neurodegenerative process in the hippocampal CA1 during the amyloid pathology of AD and that reducing brain D-serine can partially attenuate neuronal loss and reactive astrogliosis. Therefore, regulating SRR could be an effective strategy to mitigate NMDAR-dependent neurodegeneration during AD progression.

## 1. Introduction

Alzheimer’s disease (AD) is the leading cause of age-related neurodegeneration and dementia, and there is an urgent need for disease-modifying treatments given the rapidly aging population (Long and Holtzman, 2019). The neuropathological progression of AD is complex but is strongly associated with progressive extracellular deposition of amyloid β (Aβ), intracellular accumulation of neurofibrillary tangles formed by hyperphosphorylated tau protein, and ensuing induction of neuroinflammation, ultimately leading to neuronal loss and cognitive decline (DeTure and Dickson, 2019; Long and Holtzman, 2019). Although the exact mechanisms triggering AD remain unknown, Aβ accumulation is still a core pathologic criterion for AD diagnosis (Jack et al., 2018). The amyloid cascade hypothesis of AD posits that mutations in the amyloid precursor protein (APP) gene result in excessive extracellular accumulation of cytotoxic Aβ peptides (Hardy and Higgins, 1992). These soluble and insoluble Aβ oligomers induce neuroinflammatory responses, alter ionic homeostasis and transmitter uptake, and produce free radicals causing oxidative injury, ultimately activating various cell death pathways (Selkoe and Hardy, 2016).

Glutamate excitotoxicity mediated by hyperstimulation of *N*-methyl-D-aspartate receptors (NMDARs) is a major contributor to the neurodegenerative processes of AD (Parameshwaran et al., 2008; Mota et al., 2014). Extracellular Aβ oligomers have been shown to increase tonic NMDAR stimulation by disturbing glutamatergic homeostasis (Danysz and Parsons, 2012). A recent study demonstrated that Aβ dimers trigger neuronal hyperactivation by suppressing glutamate uptake, possibly by affecting glutamate transporter diffusion on astrocytes (Zott et al., 2019). Further, Aβ-induced synaptic and neuronal loss can be suppressed by NMDAR antagonists (Harkany et al., 2000; Shankar et al., 2007). Memantine, approved by the United States Food and Drug Administration for the clinical treatment of AD, is thought to function by antagonizing the NMDAR (Danysz et al., 2000; Danysz and Parsons, 2003). Memantine is a non-competitive open-channel blocker with low-affinity and rapid off-rate properties conducive to selective suppression of receptor hyperstimulation without inducing the intolerable side effects observed with other NMDAR antagonists. In fact, multiple potent NMDAR antagonists that compete with the primary agonist (glutamate) or co-agonist sites have failed to demonstrate clinically significant neuroprotection in clinical trials, mainly because of these intolerable side effects (Lipton, 2004). It is therefore essential to develop safer and more efficacious NMDAR regulators to prevent neurodegeneration in AD.

Most NMDARs are heterotetramers composed of two GluN1 and GluN2 subunits. Receptor activation requires the binding of glutamate to GluN2 subunits and either D-serine or glycine (obligatory co-agonists) to GluN1 subunits (Dingledine et al., 1999). Serine racemase (SRR), the enzyme that catalyzes the isomerization of L-serine to D-serine, is localized predominantly in neurons of the mammalian brain (Kartvelishvily et al., 2006; Miya et al., 2008; Balu et al., 2014). Thus, suppression of D-serine synthesis is a possible strategy to mitigate NMDAR-dependent neurodegeneration. Indeed, administration of the D-serine degradation enzyme D-amino acid oxidase reduced NMDAR-dependent long-term potentiation and cell death in brain slices (Yang et al., 2003; Shleper, 2005). Similarly, *Srr* gene knockout (SRRKO) mice exhibiting a 90% decrease in brain D-serine concentration showed attenuated NMDA- and Aβ-induced acute neuronal damage, supporting D-serine as a crucial mediator of Aβ-related neurotoxicity in AD (Inoue et al., 2008). In addition, several studies have reported altered D-serine levels in Aβ-injected animals, transgenic AD models, post-mortem samples of AD brain, and both cerebrospinal fluid (CSF) and serum samples from AD patients (Hashimoto et al., 2004; Madeira et al., 2015; le Douce et al., 2020; Piubelli et al., 2021). Abnormal SRR expression was also found in reactive astrocytes from AD patients and rodent models of AD (Balu et al., 2019; Folorunso et al., 2023). These discoveries suggest that dysregulation of D-serine may contribute directly to AD pathology and that modulating brain D-serine is a potential AD treatment strategy. However, the pathophysiological significance of D-serine to AD progression has not been clarified entirely.

As the precursor of D-serine and glycine, L-serine is also vital for NMDAR-mediated glutamatergic neurotransmission (Maugard et al., 2021). The biosynthesis of L-serine in the central nervous system (CNS) relies on the generation of 3-phosphoglycerate by glycolysis and subsequent conversion catalyzed by 3-phosphoglycerate dehydrogenase, phosphoserine aminotransferase, and phosphoserine phosphatase (the phosphorylated pathway) (Bridgers, 1965). Glucose hypometabolism is frequently observed in the AD brain and has been used as a diagnostic biomarker for AD (Chen and Zhong, 2013). Recent studies have also revealed alterations in the phosphorylated pathway and L-serine dysregulation during AD development (Yan et al., 2020; Chen et al., 2022; Maffioli et al., 2022). Further, disturbances in the homeostasis of other amino acids, such as alterations in alanine, aspartate, and glutamate metabolism, and imbalances among essential amino acids and short-chain acylcarnitine have been reported in patients with AD (Mahajan et al., 2020; Horgusluoglu et al., 2022). In addition, arginine biosynthesis, as well as alanine, aspartate, and glutamate metabolism, were disrupted in ordinary between AD animal models and patients with AD (Altiné-Samey et al., 2021). These pathways are highly interactive, but it is uncertain whether D-serine metabolism or signaling influences the metabolism and homeostasis of other amino acids during AD progression.

To examine the potential contributions of D-serine to AD progression, we modulated D-serine production and signaling in *App^NL–G–F/NL–G–F^* gene knock-in (APPKI) mice, an AD model expressing a humanized Aβ sequence introduced with Swedish, Iberian, and Arctic mutations (Saito et al., 2014). In contrast to APP overexpression models, APPKI mice display a higher Aβ42/Aβ40 ratio as well as amyloid plaque sizes and neuroinflammatory responses comparable to AD patients (Saito et al., 2014; Saito and Saido, 2018). In this study, we examined the associations of brain SRR and D-serine levels with age-related neuropathological changes and the effects of D-serine supplementation and *Srr* gene-deletion on AD pathology in APPKI mice. Our results suggest that D-serine can enhance excitatory neuronal damage in the hippocampal CA1 and affect neuroinflammation and amino acid homeostasis in this mouse model, while reducing D-serine can partially attenuate hippocampal neuronal death and neuroinflammation. Therefore, regulating SRR and D-serine could be a considerable NMDAR-based strategy for AD treatment.

## 2. Materials and methods

### 2.1. Animals

Male and female APPKI mice carrying the humanized *App* gene with Arctic, Swedish, and Beyreuther/Iberian mutations (Saito et al., 2014) were supplied by the RIKEN Center for Brain Science. The SRRKO mice with a C57BL/6N background were generated as previously reported (Miya et al., 2008). Wild-type (WT) C57BL/6N mice were also included as a control group in experiments and were used to expand APPKI mice. SRR deleted APPKI (APPKI-SRRKO) mice were generated by crossing APPKI mice with SRRKO mice. Genotyping of APPKI mice was conducted by polymerase chain reaction (Saito et al., 2014), and genotyping of SRRKO mice was performed using Southern blot analysis as reported (Miya et al., 2008). All mutant mice were homozygous, and only male mice were used for experiments. Animals were group-housed and maintained in a temperature- and humidity-controlled environment with free access to chow and water. All animal care and experimental procedures were performed according to the Guidelines for the Care and Use of Laboratory Animals at the University of Toyama and approved by the Ethics Committee for Animal Experiments at the University of Toyama (Permission No. A2021 MED-33). For D-serine supplementation, 0.01 M D-serine (TOCRIS, cat no. 0226, Bristol, UK) was added to the drinking water from 9 to 12 months of age.

### 2.2. Western blotting

Mice were deeply anesthetized by intraperitoneal injection of a combination of medetomidine (0.6 mg/kg body weight), midazolam (4.0 mg/kg), and butorphanol (5.0 mg/kg) (Kawai et al., 2011), and then transcardially perfused with ice-cold phosphate-buffered saline (PBS, pH 7.4). Mouse brains were removed, and the tissues from –1 mm to –3 mm relative to the bregma were cut. The hippocampal region and dorsal cortex including the retrosplenial cortex (RSC) were separated and collected for Western blotting. Samples were homogenized in ice-cold Mammalian Protein Extraction Reagent (Pierce, cat no. 78501, Rockford, IL, USA) at 0°C and centrifuged at 15,000 rpm for 15 min. The supernatants were collected and boiled with sodium dodecyl sulfate (SDS) loading buffer. Total protein extract (10 μg per gel lane) was separated by SDS-polyacrylamide gel electrophoresis and transferred onto polyvinylidene difluoride membranes. Membranes were then blocked with 5% skim milk powder in PBS for 1 h and incubated overnight at 4°C with the following primary antibodies: rabbit anti-SRR (1:2000) (Inoue et al., 2014), mouse anti-GFAP (1:2000, Sigma-Aldrich, cat no. G3893, St. Louis, MO, USA), and mouse anti-β-actin (1:2000, Sigma-Aldrich, cat no. A1349). Blotted membranes were incubated with horseradish peroxidase (HRP)-conjugated donkey anti-rabbit IgG (1:10000, Invitrogen, cat no. 31458, Carlsbad, CA, USA) or donkey anti-mouse IgG (1:10000, Invitrogen, cat no. SA1-100, Carlsbad, CA, USA) for 1 h at room temperature (RT). Targeted proteins were detected and visualized with an ECL chemiluminescence detection system (GE Healthcare, Chicago, IL, USA) and a LAS-3000 Mini Lumino image analyzer (Fujifilm, Tokyo, Japan). Protein band densities were measured using ImageJ (NIH, Bethesda, MD, USA). Band intensities were normalized to β-actin expression as the gel loading control.

### 2.3. Immunofluorescent staining

Mice were anesthetized with the same anesthetic combination described above and perfused transcardially with PBS, followed by 4% paraformaldehyde (PFA) in 0.1 M phosphate buffer (PB, pH 7.4). Mouse brains were removed, post-fixed with 4% PFA overnight, submerged in 30% sucrose in 0.1 M PB (w/v) at 4°C, frozen, and sliced into 25 μm-thick serial coronal sections using a freezing microtome. Free-floating sections were rinsed with PBS, blocked using Protein Block Serum Free (Dako, Code X0909, Carpinteria, CA, USA) for 10 min at RT, incubated at 4°C overnight with primary antibodies diluted in PBS containing 1% bovine serum albumin, and then incubated with secondary antibodies for 1 h at RT. Primary antibodies, secondary antibodies, and dilution ratios are listed in Supplementary Table 1. Sections were washed with PBS and mounted with Gold Antifade Mountant (Invitrogen, cat no. S36936, Carlsbad, CA, USA). Section images were obtained using a confocal laser scanning microscope (Leica TCS-SP5, Leica Microsystems, Mannheim, Germany) or an inverted fluorescence microscope (Keyence BZ-X800, Keyence Corporation of America, Itasca, IL, USA). The numbers of NeuN-positive cells within a 500 μm × 500 μm square area within the RSC region were quantified using ImageJ. The NeuN-positive cell numbers in hippocampal CA1 were counted manually by experimenters blinded to treatment conditions due to the high densities. Cortical amyloid plaque burdens and GFAP immunoreactive area (a measure of reactive gliosis) were also analyzed using ImageJ and normalized to the slice area.

### 2.4. Measurements of amino acid levels

Brain amino acid concentrations were measured by liquid chromatography–tandem mass spectrometry (LC/MS/MS) (Yamamoto et al., 2021). Briefly, cortical and hippocampal tissues were excised from 12-month-old mice as described in section “2.2. Western blotting,” immediately frozen in liquid nitrogen, and stored at –80°C until use. The retrieved brain tissues were ground in ice-cold 50% methanol and 50% water (30 mg tissue weight/mL) and centrifuged. The supernatant was collected into a new tube, mixed with an equal volume of chloroform, and centrifuged. The aqueous upper phase was collected, and the chloroform extraction procedure was repeated. For amino acid labeling, 50 μL of the final aqueous phase was dried and reconstituted in 50 μL LC/MS-grade water and then mixed with 10 μL of 200 mM sodium bicarbonate in water and 10 μL 1% Nα-(5-Fluoro-2,4-dinitrophenyl)-L-leucinamide (L-FDLA, TCA, cat no. A5523, Tokyo, Japan) in acetone. The mixture was incubated at 40°C for 1 h and then diluted in 930 μL 50% methanol and 50% water. Finally, 490 μL of water was added to a 10 μL sample of analyte solution and filtrated using a 0.45 μm Milex filter unit (Merck Millipore, Burlington, VT, USA). The LC/MS/MS settings were as described previously (Yamamoto et al., 2021).

Amino acid levels were calculated from the chromatographic area using Mass Hunter Quantitative analysis software (Agilent Technologies, Santa Clara, CA, USA). Contents of D- and L-serine were calculated based on standard curves constructed using the same chromatographic methods. Results are displayed as heatmaps showing individual amino acid level as a percentage of the average in WT mice. Three-dimensional principal component analysis (PCA) was performed using the data from cortical regions to reduce the dimensionality of the data set.

### 2.5. Statistical analysis

Two groups were compared by Student’s *t*-tests using Excel Statistics (Statcel 2; Social Survey Research Information Co. Ltd., Tokyo, Japan), and four groups by one-way analysis of variance (ANOVA) followed by Tukey’s *post-hoc* tests for pair-wise comparisons using Statcel 2. All data are presented as the mean ± standard error of the mean (SEM). A *p* < 0.05 was considered statistically significant for all tests (in figures, *p < 0.05, **p < 0.01, and ***p < 0.001).

## 3. Results

### 3.1. Regional alterations in SRR levels and neuronal density in APPKI mice

We first compared the expression of SRR and glial fibrillary acidic protein (GFAP) between APPKI and WT control mice. GFAP is the primary intermediate filament protein in astrocytes (Middeldorp and Hol, 2011), and its upregulation reflects astrocytic activation and neuroinflammation severity in the AD brain, which in turn is an indicator of pathological progression (Li et al., 2011; Arranz and de Strooper, 2019). Indeed, the expression of multiple GFAP isoforms were found to be increased in the brain of human AD and AD model mice (Middeldorp and Hol, 2011; Kamphuis et al., 2012).

**FIGURE 1:**
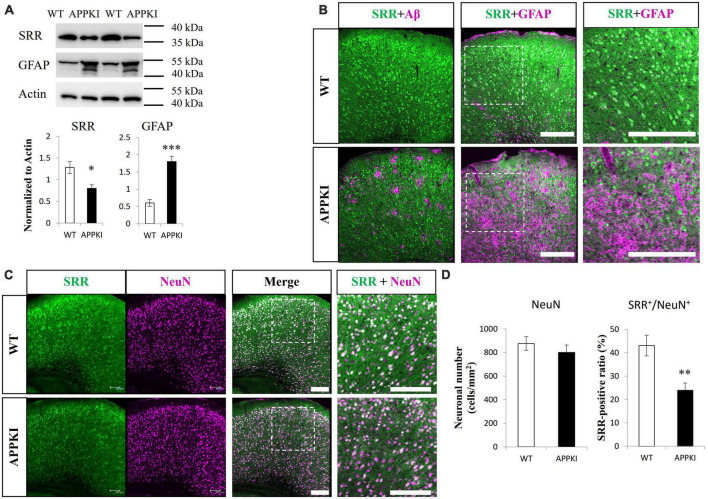
Examination of SRR expression, astrogliosis, and neuronal number in the RSC of APPKI mice. (**A** upper) Western blot analysis of SRR, GFAP, and β-actin (actin, the gel loading control) expression levels at 12 months of age. (**A** lower) Densitometric quantification of Western blot results (mean ± SEM, *n* = 4 animals per group, *p < 0.05, ***p < 0.001). **(B)** Images of RSC sections from WT (upper) and APPKI mice (lower) co-immunostained for SRR and Aβ (left) or SRR and GFAP (middle). Magnified images of the white dotted areas are shown on the right. **(C)** Co-immunostaining using anti-SRR and anti-NeuN (neuronal marker) antibodies in RSC slices from WT and APPKI mice (left, middle). Magnified images of the white dotted areas are shown on the right. (**D** left) Quantifications of average neuronal density in the RSC. (**D** right) Comparison of SRR-positive ratio (SRR^+^/NeuN^+^) in the RSC (mean ± SEM, *n* = 4 animals per group, **p < 0.01). Scale bars: 200 μm.

**FIGURE 2:**
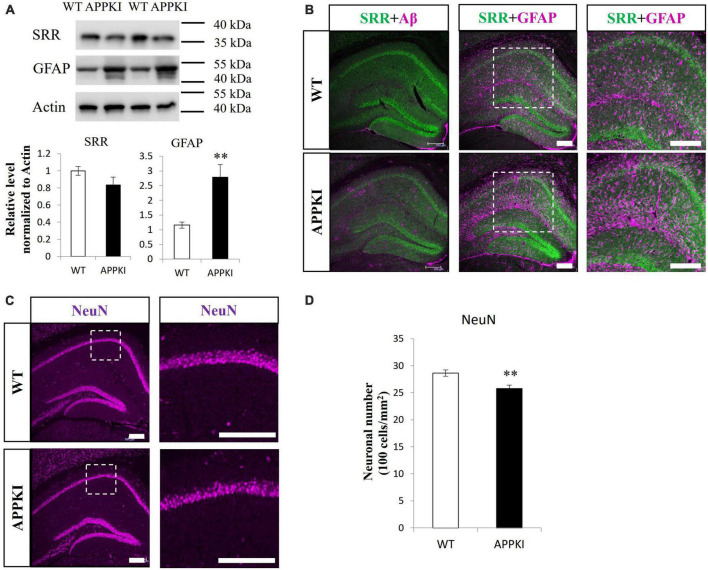
Examination of SRR expression, astrogliosis, and neuronal number in the hippocampus of APPKI mice. (**A** upper) Western blot analysis of SRR, GFAP, and β-actin (actin, gel loading control) expression levels in the hippocampus at 12 months of age. (**A** lower) Densitometric quantifications of western blot results (mean ± SEM, *n* = 4 animals per group, **p < 0.01). **(B)** Images of the hippocampus from WT (upper) and APPKI mice (lower) co-immunostained for SRR and Aβ (left) or SRR and GFAP (middle). Magnified images of the white dotted areas are shown on the right. **(C)** Immunostaining for NeuN in hippocampal slices from WT and APPKI mice (left). Magnified images of the white dotted areas are shown on the right. **(D)** Quantifications of average neuronal density in the hippocampal CA1 region (mean ± SEM, *n* = 4 animals per group, **p < 0.01). Scale bars: 200 μm.

Serine racemase (SRR) levels were significantly reduced in the RSC of APPKI mice at 12-month-old compared with age-matched WT mice as estimated by Western blotting (*p* < 0.05, Figure 1A). In accordance with enhanced astrogliosis observed in other AD animal models and post-mortem tissues from AD patients (Verkhratsky et al., 2019), APPKI mice also exhibited significantly greater GFAP expression with a lower molecular weight band at 12 months in RSC compared with age-matched WT mice (*p* < 0.001, Figure 1A). Hippocampal SRR expression was slightly lower in 12-month-old APPKI mice but not significantly (*p* = 0.17, Figure 2A), while hippocampal GFAP level was markedly higher compared to that of age-matched WT mice (*p* < 0.01, Figure 2A). Immunofluorescence results revealed qualitatively similar regional changes in SRR and GFAP expression at 12 months of age (Figure 1B and Figure 2B). The SRR signals were observed primarily in neurons but were hardly detected in GFAP-positive cells in the RSC of 12-month-old APPKI mice (Figures 1B, C). To investigate whether this SRR reduction in RSC was due to neuronal loss concomitant with AD progression, we examined the association between neuronal number and SRR expression by co-immunostaining for the neuron-specific protein NeuN and SRR. Numbers of NeuN-positive cells were comparable in the RSC of APPKI and WT mice, but the ratio of SRR-positive to NeuN-positive cells was significantly reduced in APPKI mice (*p* < 0.01, Figure 1D), suggesting a cell type-specific downregulation of SRR expression in the RSC. In contrast to the RSC, the number of NeuN-positive cells in the hippocampal CA1 was significantly reduced in 12-month-old APPKI mice compared with age-matched control mice (*p* < 0.01, Figures 2C, D), suggesting greater neurodegenerative progression in the hippocampus of APPKI mice.

### 3.2. D-Serine supplementation exacerbated neuronal loss in the hippocampal CA1 but not RSC of APPKI mice

**FIGURE 3:**
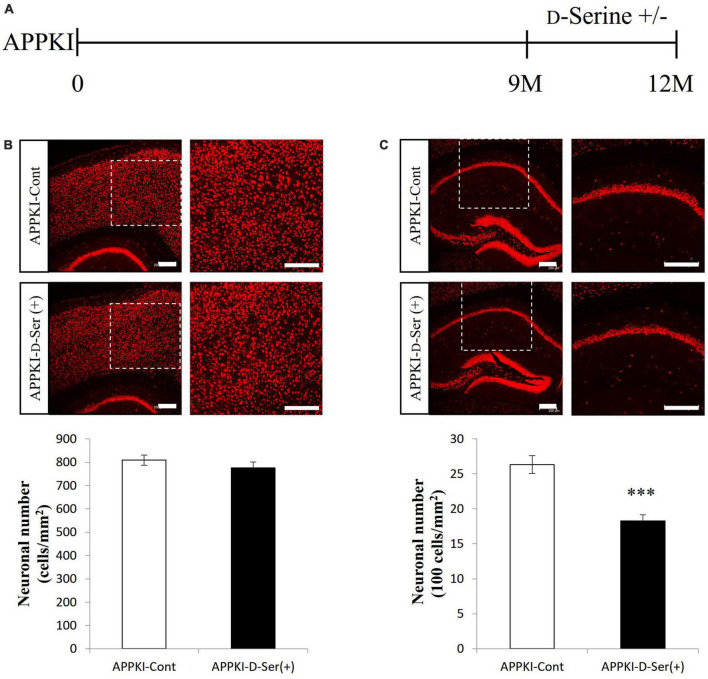
D-Serine supplementation in drinking water exacerbated neuronal loss in the hippocampal CA1 but not RSC of APPKI mice. **(A)** Procedure of D-serine supplementation. Drinking water without D-serine (D-Serine —) or with D-serine (D-Serine +, 0.01 M) was given to APPKI mice from 9 to 12 months of age. (**B,C** upper) Representative images of cortical **(B)** and hippocampal **(C)** slices from APPKI mice receiving only drinking water [APPKI-Cont] or D-serine supplementation [APPKI-D-Ser(+)] (left). Magnified images of the white dotted areas are shown on the right. (**B,C** lower) Quantification of average neuronal density in the cortex **(B)** and hippocampus **(C)** of [APPKI-Cont] and [APPKI-D-Ser(+)] mice (mean ± SEM, *n* = 4 animals per group, ***p < 0.001). Scale bars: 200 μm.

Both WT and APPKI mice were administered D-serine in drinking water from 9 to 12 months of age (Figure 3A and Supplementary Figure 1A), and effects on AD-like neurodegeneration were evaluated. The D-serine concentration (0.01 M) was chosen based on a previous report (Tabata-Imai et al., 2014). Three months of D-serine supplementation enhanced neuronal loss in the hippocampal CA1 region of APPKI mice but not the RSC of APPKI mice or the hippocampal CA1 of WT mice (*p* < 0.001, Figures 3B, C and Supplementary Figures 1B, C). In APPKI mice receiving D-serine supplementation, both D-serine and L-serine levels in the cortex were increased significantly compared to mice receiving only water (Supplementary Figure 2). These findings suggest that excessive brain D-serine can indeed exacerbate NMDAR-dependent neurotoxicity in the hippocampal CA1 of APPKI mice. Furthermore, these results suggest that hippocampal neurons are more vulnerable to exogenous D-serine than RSC neurons. There were no significant changes in cortical SRR and GFAP levels in APPKI mice receiving D-serine supplementation at 12 months of age (Supplementary Figure 3).

### 3.3. *Srr* gene-deletion attenuated astrogliosis and hippocampal neuronal damage in APPKI mice

**FIGURE 4:**
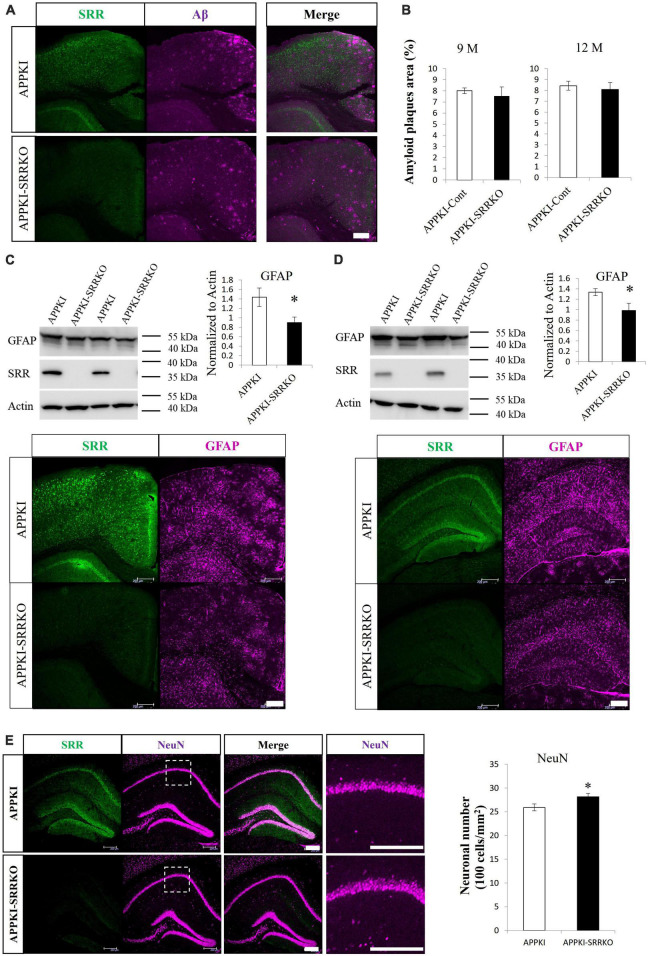
*Srr* gene-deletion attenuated the pathological changes in APPKI mice. **(A)** Co-immunostaining for SRR and Aβ in cortical slices from APPKI (upper) and APPKI-SRRKO (lower) mice at 9 months of age. **(B)** Comparisons of amyloid plaque burden in the RSC between APPKI and APPKI-SRRKO mice at 9 months of age (left) and 12 months of age (right) (mean ± SEM, *n* = 4 animals per group). (**C,D** upper) Western blot analysis of the cortical (**C** left) and hippocampal (**D** left) tissues from 9-month-old APPKI and APPKI-SRRKO mice probed with antibodies against GFAP, SRR, and β-actin (actin, gel loading control). Quantification of GFAP protein expression normalized to actin (right, mean ± SEM, *n* = 6 animals per group, *p < 0.05). (**C,D** lower) Images of slices from the cortex **(C)** and hippocampus **(D)** of 9-month-old APPKI (upper) and APPKI-SRRKO (lower) mice co-stained with anti-SRR and anti-GFAP antibodies. (**E** left) Images of hippocampal slices co-immunostained with anti-SRR and anti-NeuN antibodies from 12-month-old APPKI (upper) and APPKI-SRRKO (lower) mice. Magnified images of dotted areas are also shown. (**E** right) Quantification of NeuN-positive cell number in the hippocampal CA1 region (mean ± SEM, *n* = 6 animals per group, *p < 0.05). Scale bars: 200 μm.

To provide further evidence for the contribution of endogenous D-serine to AD progression and the potential of AD treatment by SRR regulation, we generated APPKI-SRRKO mice by crossing APPKI mice with SRRKO mice and examined effects on AD pathology. Immunostaining confirmed ablation of SRR expression in APPKI-SRRKO mice, but there was no significant difference in amyloid plaque levels in the cortex between APPKI and APPKI-SRRKO mice at 9 and 12 months of age (Figures 4A, B). However, 9-month-old APPKI-SRRKO mice exhibited lower GFAP levels than age-matched APPKI mice in both cortical and hippocampal tissues as measured by Western blotting (both *p* < 0.05, Figures 4C, D). Furthermore, GFAP-immuno-positive area was significantly reduced in 9-month-old APPKI-SRRKO mice compared with age-matched APPKI mice (*p* < 0.001, Supplementary Figure 4), suggesting that endogenous D-serine can also affect astrogliosis and neuroinflammation during the amyloid pathology in APPKI mice. The number of NeuN-positive cells was also significantly higher in the hippocampal CA1 region of 12-month-old APPKI-SRRKO mice compared with age-matched APPKI mice (*p* < 0.05, Figure 4E), suggesting that suppressed D-serine synthesis in the AD brain can reduce hippocampal neuronal damage. These results are in accordance with the finding that D-serine supplementation promotes neuronal damage in the hippocampus of APPKI mice (Figure 3) and strongly suggest that endogenous D-serine participates in NMDAR-dependent excitatory neurotoxicity in the hippocampal CA1 during the amyloid pathology of AD.

### 3.4. *Srr* gene-deletion partially reversed altered cortical amino acid homeostasis in APPKI mice

**FIGURE 5:**
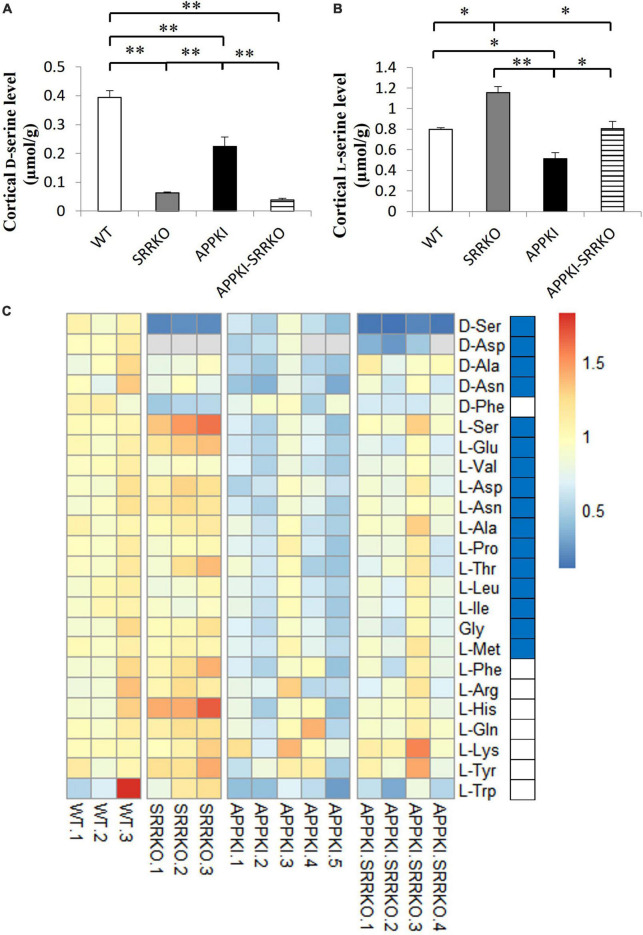
*Srr* gene-deletion partially reversed the abnormal amino acid profiles in the cortex of APPKI mice. **(A)** Analysis of D-serine concentrations in cortical tissues from WT, SRRKO, APPKI, and APPKI-SRRKO mice (mean ± SEM, *n* = 3, 3, 5, and 4 mice in each group, respectively; **p < 0.01). **(B)** Analysis of L-serine concentrations in cortical tissues from WT, SRRKO, APPKI, and APPKI-SRRKO mice (*n* = 3, 3, 5, 4 mice in each group, respectively; *p < 0.05, **p < 0.01). **(C)** Heatmap of 24 amino acids detected in the RSC from WT, SRRKO, APPKI, and APPKI-SRRKO mice. Rows represent amino acids and columns represent individual mice. Levels of all amino acids were normalized to the corresponding mean value in the WT group. The different colors depict the relative levels of each amino acid. Red and blue colors represent upregulation and downregulation, respectively. Blue blocks beside the heatmap indicate significantly decreased amino acids in APPKI mice compared with WT mice (by one-tailed *t*-test). D-Aspartate concentrations were below the detection limit in SRRKO mouse No. 1–3, APPKI mouse No. 4 and 5, and APPKI-SRRKO mouse No. 4 (gray).

Finally, we examined if Aβ pathology and brain D-serine manipulation can alter amino acid concentration profiles using LC/MS/MS (Yamamoto et al., 2021). As expected, D-serine concentration was significantly reduced in both the hippocampus and cortex of 12-month-old SRRKO and APPKI-SRRKO mice compared with age-matched WT and APPKI mice (all *p* < 0.01, Figure 5A and Supplementary Figure 5A), demonstrating that *Srr* gene-knockout effectively suppresses D-serine synthesis throughout the forebrain. In the cortical region, APPKI mice exhibited decreased D-serine levels compared with WT mice at 12-month-old (*p* < 0.01, Figure 5A), which coincides with decreased SRR expressions in APPKI mice, as shown in Figure 1A. Surprisingly, L-serine concentration was also reduced in APPKI mice compared to the other three groups (*p* < 0.01, Figure 5B), indicating that the decline in D-serine content was due to the deficits in both SRR expression and L-serine metabolism. L-Serine concentration in SRRKO mice was higher than in the other three groups (*p* < 0.05 or *p* < 0.01, Figure 5B), probably because of the deficiency in producing D-serine from L-serine. Similarly, L-serine was significantly higher in APPKI-SRRKO mice than in APPKI mice and comparable to WT mice (Figure 5B). Relative levels of all detected amino acids in each mouse are shown in the heatmap (Figure 5C). We also observed significant reductions in the concentrations of L-Glu, L-Val (both *p* < 0.01), L-Asp, L-Asn, L-Ala, L-Pro, L-Thr, L-Leu, L-Ile, Gly, L-Met (all *p* < 0.05), D-Asp, D-Asn (both *p* < 0.05), and D-Ala (*p* < 0.01) in the RSC of APPKI mice compared with WT mice (all comparisons by one-tailed Student’s *t*-test), but no significant differences in L-Phe, L-Arg, L-His, L-Gln, L-Lys, L-Tyr, and L-Trp (Figure 5C). Some of these reductions were partially reversed in APPKI-SRRKO mice (Figure 5C). Then, we used three-dimensional PCA to simplify the complexity of the high-dimensional original dataset and bring out a strong pattern from it (Lever et al., 2017). In simple, the data of amino acid levels from each mouse were condensed into one single dot in a three-dimensional space that represents the amino acid profile of that mouse. Individuals with similar amino acid profiles cluster together, while those with larger gaps in profiles are farther apart in space. With principal component (PC) 1 revealing the most variation and PC2 and PC3 representing the second and third most variation in the data, distances among individuals or clusters along the PC1 axis show the most significant differences in amino acid profiles. We found that PCA revealed notable differences in variance among groups (Supplementary Figure 6). PC1 captured 67.1% of the variations in amino acid concentrations, while PC2 and PC3 captured 14.9 and 5.1% of the variances. The amino acid concentration profile of the APPKI mouse brain showed the most remarkable differences from WT mice along PC1, and these differences were partially mitigated in APPKI-SRRKO mice, indicating that *Srr* gene-deletion can partially restore normal amino acid homeostasis in the cortex of APPKI mice. In contrast, we did not detect significant differences in hippocampal D-serine and L-serine concentrations between APPKI and WT mice (Supplementary Figures 5A, B). Hippocampal concentrations of most amino acids were also indistinguishable among groups except for D-type amino acids due to the variability of individuals (Supplementary Figure 5C).

## 4. Discussion

### 4.1. D-Serine and AD progression

In the current study, we observed that SRR and D-serine levels were significantly reduced in the RSC of APPKI mice compared with WT mice at 12 months of age (Figure 1A and Figure 5A). The ratio of SRR-positive neurons was also reduced in the RSC of APPKI mice (Figure 1D), indicating that some cortical neurons lose SRR expression in APPKI mice.

The amyloid plaque burden increased in the cortex and hippocampus of APPKI mice from 3 to 9 months of age, whereas no significant difference was found between 9 and 12 months of age, as reported previously (Mehla et al., 2019). We also confirmed that amyloid deposits peak around 9-month-old in APPKI mice (Figure 4B). However, the balance between soluble and insoluble Aβ oligomers may continue to change, which may lead to subsequent pathological changes, and altered SRR expression.

We also found that exogenous D-serine supplementation can exacerbate neuronal loss in the hippocampal CA1 but not the RSC of APPKI mice (Figure 3). Previous studies have demonstrated that the NMDAR co-agonist binding sites in the hippocampus are not saturated under baseline (Wood, 1995; Li et al., 2009), remaining accessible to exogenous D-serine, thus exacerbating NMDAR hyperactivation. Conversely, these binding sites may be saturated in the RSC, therefore preventing the impact of exogenous D-serine supplementation. In addition, some NMDAR antagonists can induce neurotoxicity in specific regions, including RSC, which may depend on the disinhibition of pyramidal neurons by antagonizing NMDARs on inhibitory interneurons in these certain areas (Olney et al., 1989, 1991; Li et al., 2002). These studies suggest that disinhibition may be an alternative mechanism underlying neurotoxicity in the RSC. Hence, hippocampal neurons were more vulnerable to excessive D-serine supplementation than RSC neurons in APPKI mice. D-Serine may not be the origin of neurodegeneration because there were no changes in SRR and D-serine levels but neuronal losses in the hippocampus. However, it is worth mentioning that *Srr* gene-deletion can partially attenuate neuronal damage in the hippocampal CA1 of APPKI mice (Figure 4E), suggesting that D-serine participates in hippocampal neuronal damage during AD amyloid pathology.

The NMDAR mediates a major proportion of glutamatergic transmission in the mammalian CNS and is required for many forms of use-dependent synaptic plasticity linked to learning and memory (Mori, 2017). However, excessive NMDAR stimulation results in excitotoxicity. Because D-serine is a necessary co-agonist for NMDAR activation, a high brain D-serine level is expected to exacerbate excitotoxicity in neurodegenerative diseases such as AD. Indeed, elevated D-serine levels were found in rodent brains following Aβ injection as well as in various transgenic AD models, post-mortem brain samples from AD patients, and both CSF and serum samples from AD patients (Madeira et al., 2015; Piubelli et al., 2021), suggesting that elevation of D-serine contributes to excitatory neurotoxicity in AD; however, other studies have found inconsistent results (Hashimoto et al., 2004; Biemans et al., 2016; Nuzzo et al., 2020). Moreover, promotion of NMDAR-dependent synaptic plasticity by D-serine supplementation has been found to improve cognitive impairment in 3xTg AD mice reportedly caused by insufficient glycolysis-derived L-serine (le Douce et al., 2020). Conversely, a recent study reported that the expression of L-serine-producing enzyme phosphoglycerate dehydrogenase was increased in the hippocampus of two AD mouse models and brain samples from advanced AD patients (Chen et al., 2022). Therefore, the efficacy and safety of dietary L- or D-serine supplementation for AD treatment are uncertain, especially as brain D-serine may display complex concentration changes and different roles during the long AD progression (Ni and Mori, 2022). We suggest that D-serine may improve cognitive impairment in the early phase of AD (le Douce et al., 2020) and promote the survival of newborn neurons (Sultan et al., 2013). However, in the late phases of AD, D-serine may aggravate AD pathology by enhancing excitotoxicity and neuroinflammation.

### 4.2. D-Serine and astrogliosis in AD

Several studies have reported abnormal glial expression of SRR in neurodegenerative diseases associated with neuroinflammation (Sasabe et al., 2007; Perez et al., 2017; Balu et al., 2019; Folorunso et al., 2023). However, we did not detect enhanced expression of astrocytic SRR in APPKI mice at 12-month-old. Astrocytic SRR was observed in the brain of other AD models, including the amygdala of 5xFAD mice and the hippocampus and cortex of TgF344-AD rats, as reported (Balu et al., 2019; Folorunso et al., 2023). Except for mutated form of human *APP* genes, these AD models also express mutated presenilin 1 transgene, while APPKI mice only carry human *APP* gene mutations. Moreover, the appearance of astrocytic SRR was observed in aged but not young AD models, when robust reactive astrogliosis exists, suggesting that mouse lines and disease stages may affect the onset of astrocytic SRR expression. Conversely, the NMDAR blocker memantine inhibited glial activation, suggesting that neuroinflammation is, in part, NMDAR-dependent (Wu et al., 2009; Sühs et al., 2016). Memantine administration also reduced gliosis in APP/PS1 Tg mice as well as in rats following ibotenic acid infusion (Ahmed et al., 2004; Scholtzova et al., 2008). Although D-serine supplementation did not enhance astrogliosis in APPKI mice at 12 months of age (Supplementary Figure 3), possibly due to a ceiling effect, APPKI-SRRKO mice exhibited lower GFAP expression than APPKI mice at 9 months of age (Figures 4C, D and Supplementary Figure 4), again suggesting that D-serine influences astrogliosis during AD progression by promoting excessive NMDAR activity.

### 4.3. Interactions between D-serine and other amino acids in AD

The APPKI mice also demonstrated broad changes in cortical amino acid concentrations compared with WT mice (Figure 5C). Based on the Kyoto Encyclopedia of Genes and Genomes pathway database,^1^ our results infer that alanine, aspartate, and glutamate metabolism pathway, as well as glycine, serine, and threonine metabolism pathway, are disrupted in the cortex of APPKI mice. Previous metabolomic analyses also revealed changes in alanine, aspartate, and glutamate metabolism and arginine biosynthesis in AD models and patients (Altiné-Samey et al., 2021), suggesting that disturbances in amino acid metabolism are involved in AD pathogenesis.

Glucose uptake and metabolism are known to be impaired during AD progression, probably due to oxidative damage of glycolytic proteins, leading to decreased energy generation (Butterfield and Halliwell, 2019). The posterior cingulate cortex and the RSC were found to exhibit hypometabolism in early AD (Minoshima et al., 1997; Nestor et al., 2003). This hypoactivity may in turn disrupt the activities of other regions anatomically associated with the RSC, such as the hippocampal formation. Thus, RSC hypoactivity may contribute to memory loss in patients with AD (Vann et al., 2009). To maintain sufficient energy generation, neurons in the AD brain may rely on other sources, such as amino acids and ketone bodies, leading to metabolite disturbances (Griffin and Bradshaw, 2017), which may partially explain the amino acid dyshomeostasis observed in the RSC of APPKI mice.

Furthermore, levels of some essential amino acids, especially branched-chain amino acids (Val, Leu, Ile), are reduced in the cortex of APPKI mice (Figure 5C), possibly due to altered transport. For instance, abnormalities in essential amino acid levels have been reported in the plasma of APPswe/PS1deltaE9 double transgenic AD model mice (Pan et al., 2016). Also, a meta-analysis of prospective cohort studies concluded that lower blood levels of branched-chain amino acids are related to a higher risk of dementia and AD (Tynkkynen et al., 2018). However, the etiologic role of branched-chain amino acids in AD requires further investigation.

Abnormalities in some cortical amino acid concentrations were partially reversed in APPKI-SRRKO mice (Figure 5C and Supplementary Figure 6), indicating that D-serine signaling or metabolism can affect the homeostasis of other amino acids during AD progression. Oxidative stress contributes to dysfunctional metabolism, so suppressing D-serine may maintain normal amino acid homeostasis by inhibiting oxidative damage induced by NMDAR hyperactivation in AD brains (Kamat et al., 2016). Besides, *Srr* gene-deletion in APPKI-SRRKO mice restored L-serine levels (Figure 5B), which may also conduce to amino acid homeostasis recovery. These current findings suggest that D-serine and SRR activity may influence amino acid metabolism and the transport of amino acids across the blood-brain barrier.

### 4.4. Limitation of this study

Females were affected by AD more severely, and gender may interact with other AD risk factors to progress the disease process (Udeh-Momoh and Watermeyer, 2021). Numerous factors may contribute to heightened sensitivity to AD in females, such as gender-associated differences in microglia. Sex differences in amyloidogenesis and microglial activation were also observed in APPKI mice. Female APPKI mice displayed faster progression of microglial activation and higher plaque burdens (Masuda et al., 2016; Sala Frigerio et al., 2019; Biechele et al., 2020). A recent multi-omics analysis in AD patients showed that serine metabolism and D-serine/total serine ratio were significantly altered and exhibited gender differences during AD progression (Maffioli et al., 2022), indicating distinct pathophysiological mechanisms in males and females. Only male mice were used for the current experiments, whereas dynamic changes of D-serine and its related pathway may be modulated differently in female mice. Moreover, amyloid depositions and Aβ-related neuroinflammation were observed in APPKI mice, but neurofibrillary tangles were hardly found in APPKI mice and other *APP* transgenic mice (Sasaguri et al., 2017), which means that APPKI mice only partially replicate AD pathology. It is still unclear whether tauopathy affects SRR expression and localization.

## 5. Conclusion

We observed decreased D-serine and SRR levels in the RSC during the pathophysiological progression in APPKI mice. In addition, long-term, large-dose D-serine supplementation exacerbated neuronal damage in the hippocampal CA1 but not in the RSC of APPKI mice, while reducing brain D-serine concentration by *Srr* gene-deletion attenuated neuronal damage in the hippocampal CA1 and astrogliosis. These findings reveal that (1) D-serine participates in the regional neurodegenerative process in the hippocampal CA1 during the amyloid pathology of AD and that (2) hippocampal neurons are more susceptible to exogenous D-serine supplementation than cortical neurons. Moreover, amino acid dyshomeostasis in the cortex of APPKI mice was partially reversed by *Srr* gene-deletion, indicating that D-serine signaling or metabolism influences the homeostasis of other amino acids, which in turn may contribute to AD progression. Taken together, these findings suggest that reducing brain SRR activity and/or D-serine may be an effective method for practicing NMDAR-targeted strategy in AD treatment. Further investigations are required to determine if D-serine changes dynamically during different AD phases or in different brain regions and if these changes are gender-related. Such knowledge is essential for the clinical use of D-serine or SRR modulators for AD treatment.

## Data availability statement

The original contributions presented in this study are included in the article/Supplementary material, further inquiries can be directed to the corresponding author.

## Ethics statement

The animal study was reviewed and approved by the Ethics Committee for Animal Experiments at the University of Toyama.

## Author contributions

XN, RI, and HM designed the experiments and wrote and edited the manuscript. XN, RI, and YW performed experiments and analyzed and interpreted the data. TY, KY, and TN provided technical support. TS, TCS, and KT provided experimental animals. All authors contributed to the article and approved the submitted version.

## Footnotes

1
https://www.kegg.jp/
