# Supplementary material for: Regional contributions of D-serine to Alzheimer’s disease pathology in male AppNL–G–F/NL–G–F mice
Source: Front Aging Neurosci. 2023 Jun 29;15:1211067. doi: 10.3389/fnagi.2023.1211067 (PMC10339350; doi:10.3389/fnagi.2023.1211067)
Supplement: Supplementary file 1 [file Data_Sheet_1.docx]

Supplementary Material

**Regional contributions of D-serine to Alzheimer’s disease pathology in male *App*^NL-G-F/NL-G-F^ mice**

Authors: Xiance Ni ^1, 2†^, Ran Inoue ^1, 3†^, Yi Wu ^1, 2^, Tomoyuki Yoshida ^1, 3^, Keisuke Yaku ^4^, Takashi Nakagawa^4, 5^, Takashi Saito ^6, 7^, Takaomi C Saido ^6^, Keizo Takao ^3, 5, 8^, Hisashi Mori ^1, 3, 5 *^

*** Correspondence:**

Hisashi Mori: [hmori@med.u-toyama.ac.jp](javascript:%20addrSendMail('%25E6%25A3%25AE%2520%25E5%25AF%25BF',%20'hmori%2540med.u-toyama.ac.jp');)

^†^These authors have contributed equally to this work and share first authorship.

1. Department of Molecular Neuroscience, Faculty of Medicine, University of Toyama, Toyama, Japan.

2. Graduate School of Innovative Life science, University of Toyama, Toyama, Japan.

3. Research Center for Idling Brain Science (RCIBS), University of Toyama, Toyama, Japan.

4. Department of Molecular and Medical Pharmacology, Faculty of Medicine, University of Toyama, Toyama, Japan.

5. Research Center for Pre-Disease Science, University of Toyama, Toyama, Japan.

6. Laboratory for Proteolytic Neuroscience, RIKEN Center for Brain Science (CBS), Saitama, Japan.

7. Department of Neurocognitive Science, Institute of Brain Science, Nagoya City University Graduate School of Medical Sciences, Aichi, Japan.

8. Department of Behavioral Physiology, Faculty of Medicine, University of Toyama, Toyama, Japan.

Supplementary Figure 1


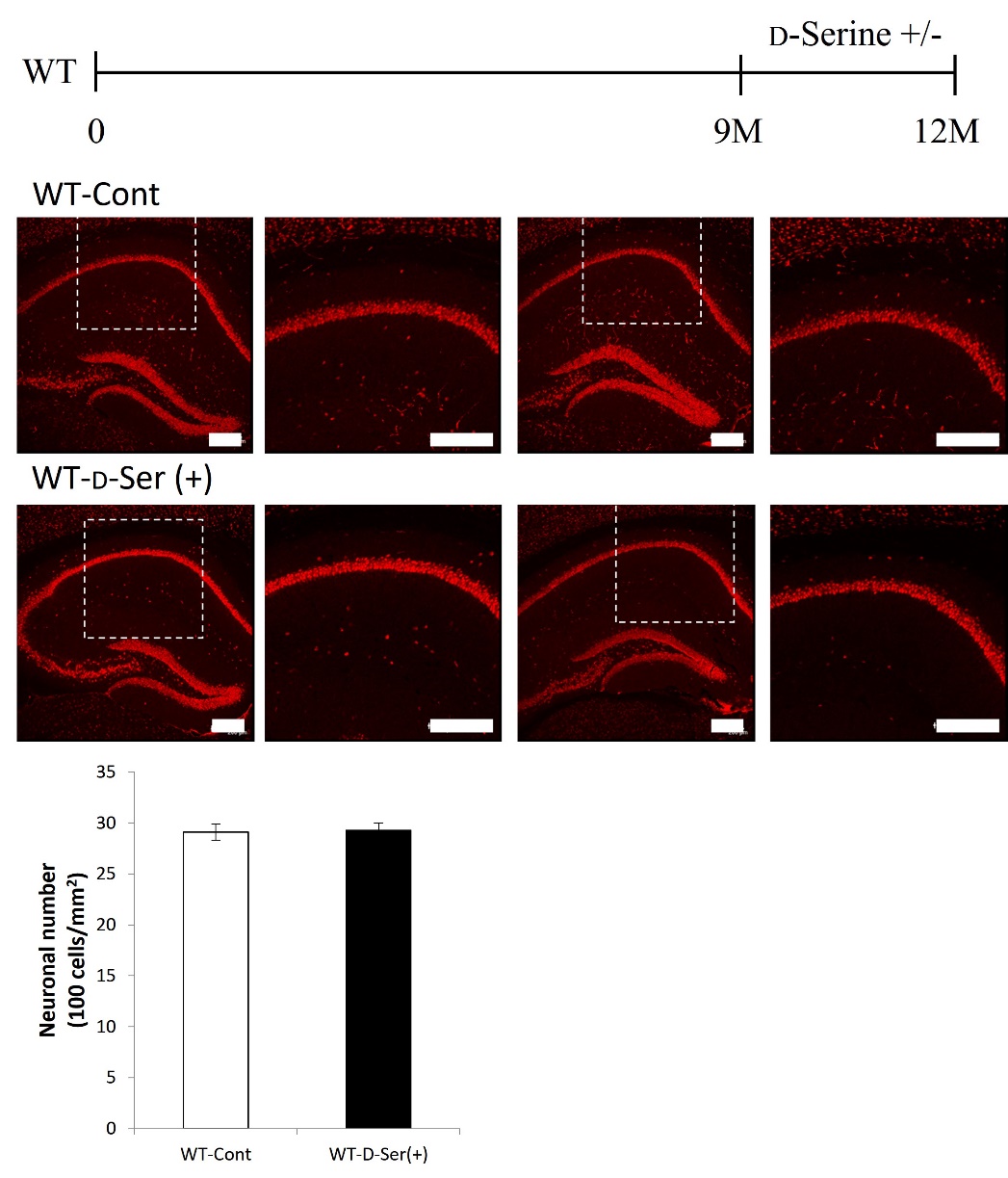


Cv

Bv

Av

Supplementary Figure 1. Effect of D-serine supplementation on neuronal number in WT mice.

(A) Procedure of D-serine supplementation. (B) Representative images of hippocampal slices stained with anti-NeuN antibody from WT mice receiving only water (WT-Cont) or D-serine supplementation (WT-D-Ser (+)). Magnified images of the dotted areas are also shown. (C) Average neuronal numbers in hippocampal CA1 of WT-Cont and WT-D-Ser (+) mice (mean ± SEM, n = 4 animals per group). Scale bars: 200 µm.

Supplementary Figure 2


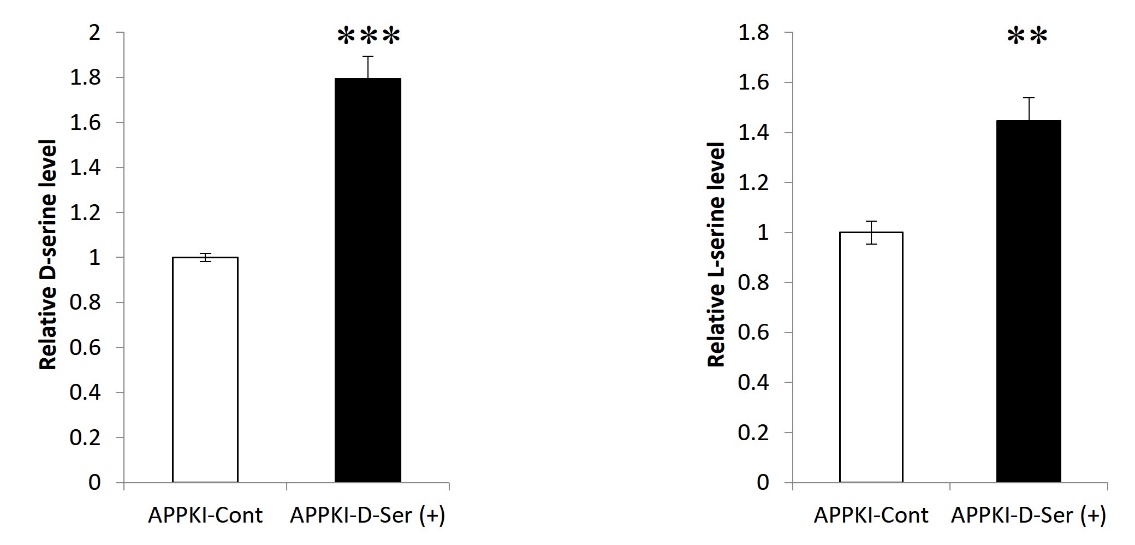


Bv

Av

Supplementary Figure 2. Relative D- and L-serine levels in the cortices of APPKI mice receiving only water and three months of D-serine supplementation.

(A) Relative D-serine levels in APPKI mice receiving only water (APPKI-Cont) and D-serine supplementation (APPKI-D-Ser (+)). (B) Relative L-serine levels in APPKI mice receiving only water (APPKI-Cont) and D-serine supplementation (APPKI-D-Ser (+)) (mean ± SEM, n = 4 animals per group, **p < 0.01, ***p < 0.001).

Supplementary Figure 3


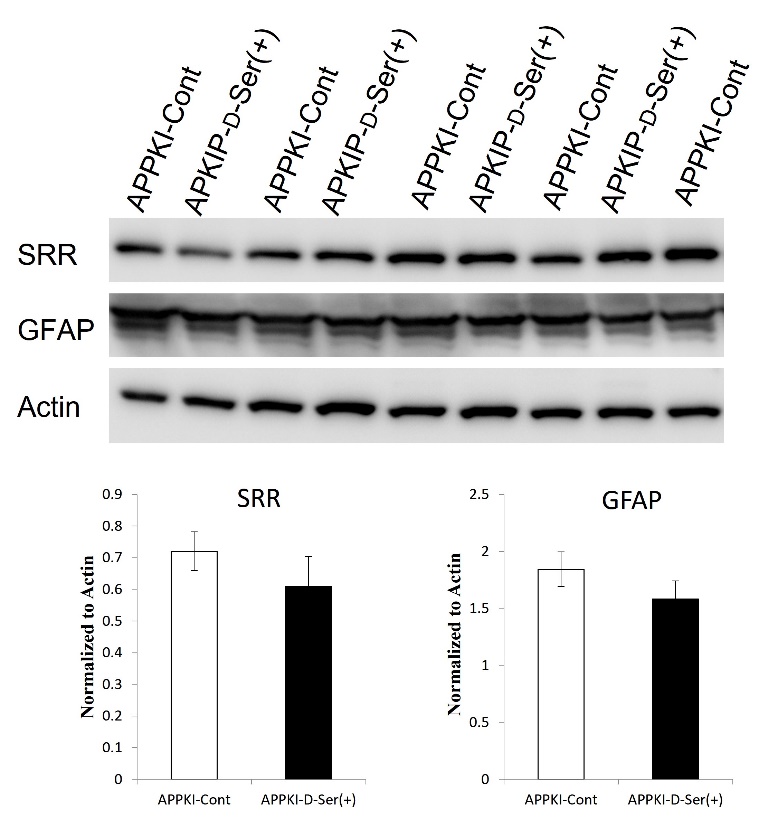


Cv

Bv

Av

Supplementary Figure 3. SRR and GFAP levels in APPKI mice receiving D-serine supplementation for 3 months.

(A) Western blot analysis of cortical SRR and GFAP expressions in APPKI mice receiving only water (APPKI-Cont) or D-serine supplementation (APPKI-D-Ser (+)). β-Actin (Actin) was used as the gel loading control. (B, C) Quantifications of SRR and GFAP levels normalized to Actin expression (mean ± SEM, n = 5 animals from the APPKI-Cont group and n = 4 animals from the APPKI-D-Ser (+) group).

Supplementary Figure 4

Av


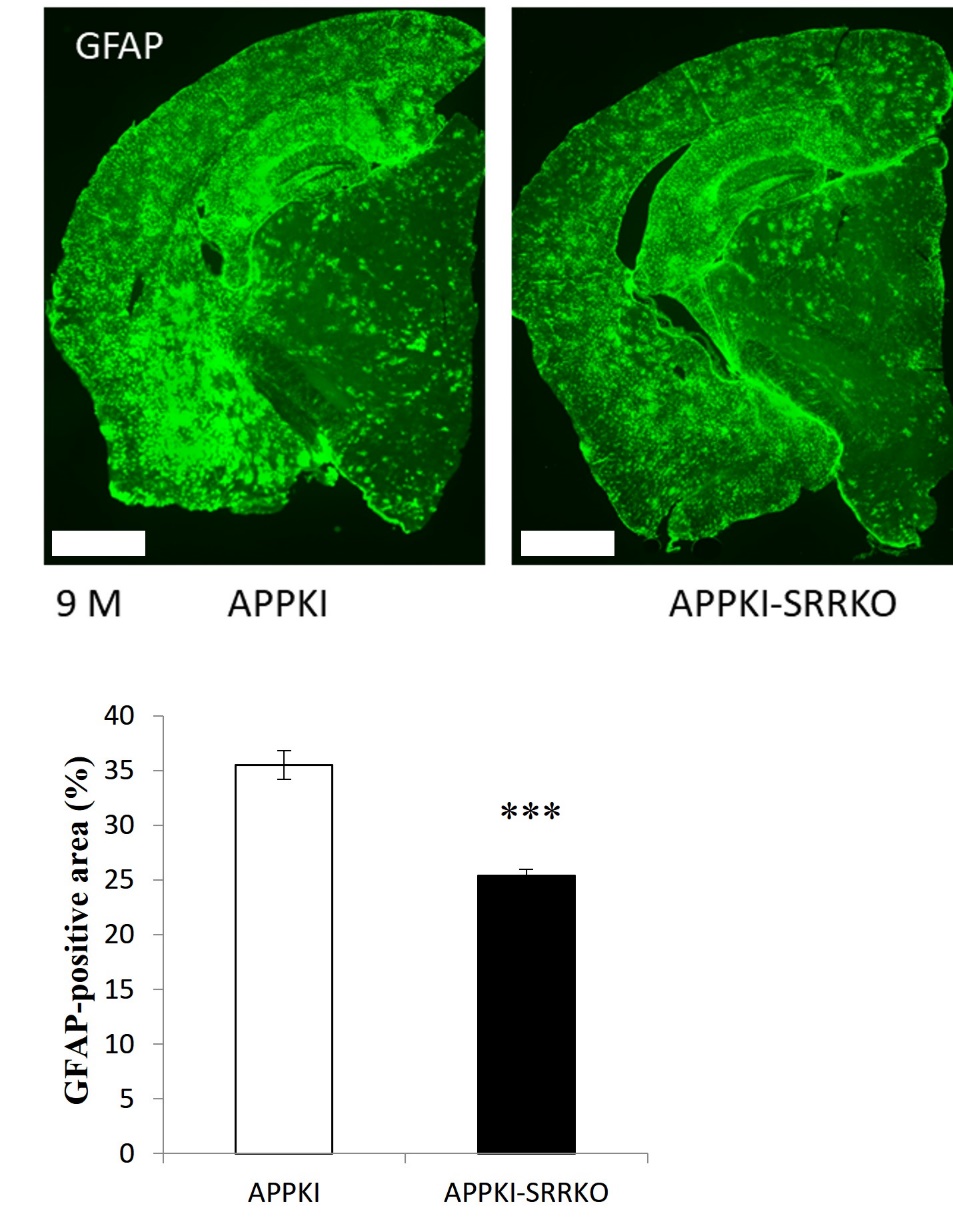


Bv

Supplementary Figure 4. GFAP staining of slices from APPKI and APPKI-SRRKO mice.

(A) Slices from 9-month-old APPKI (left) and APPKI-SRRKO (right) mice stained with anti-GFAP antibody. (B) Quantification of GFAP-positive area ratio (GFAP-positive area/whole slice area) in APPKI and APPKI-SRRKO mice (mean ± SEM, n = 3 animals per group, ***p<0.001). Scale bars: 1 mm.

Supplementary Figure 5


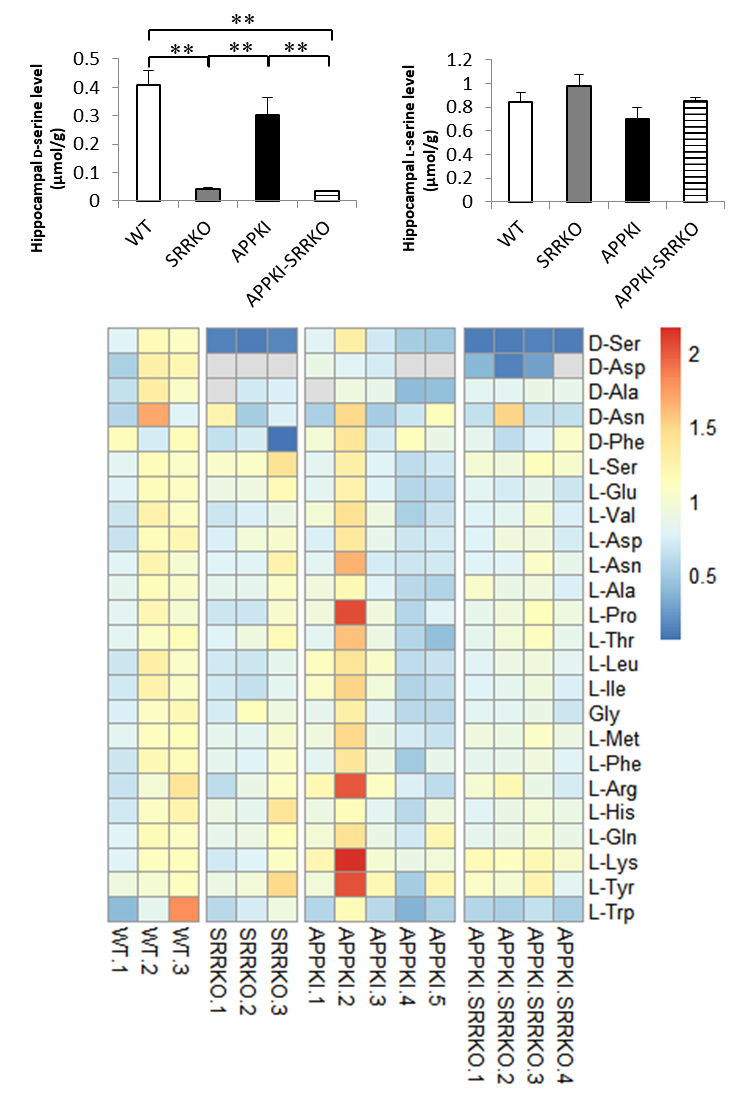


Av

Bv

Cv

Supplementary Figure 5. Amino acid profiling in the hippocampus.

(A) Analysis of D-serine concentrations in hippocampal tissues from WT, SRRKO, APPKI, and APPKI-SRRKO mice (mean ± SEM, n = 3, 3, 5, 4 mice in each group, respectively; **p<0.01). (B) Analysis of L-serine concentrations in hippocampal tissues from WT, SRRKO, APPKI, and APPKI-SRRKO mice (mean ± SEM, n = 3, 3, 5, 4 mice in each group, respectively). (C) Heatmap of 24 amino acids detected in the hippocampus of WT, SRRKO, APPKI, and APPKI-SRRKO mice. Rows represent amino acids and columns represent individual mice. Levels of each amino acid were normalized to the mean value of the corresponding amino acid in WT mice. The different colors of the heatmap depict the relative level of each amino acid. Red and blue colors represent upregulation and downregulation of amino acid levels, respectively. The levels of D-aspartate in SRRKO mouse No. 1–3, APPKI mouse No. 4 and 5, and APPKI-SRRKO mouse No. 4, and the levels of D-alanine in SRRKO mouse No.1 and APPKI mouse No.1 were below the detection limit (grey).

Supplementary Figure 6


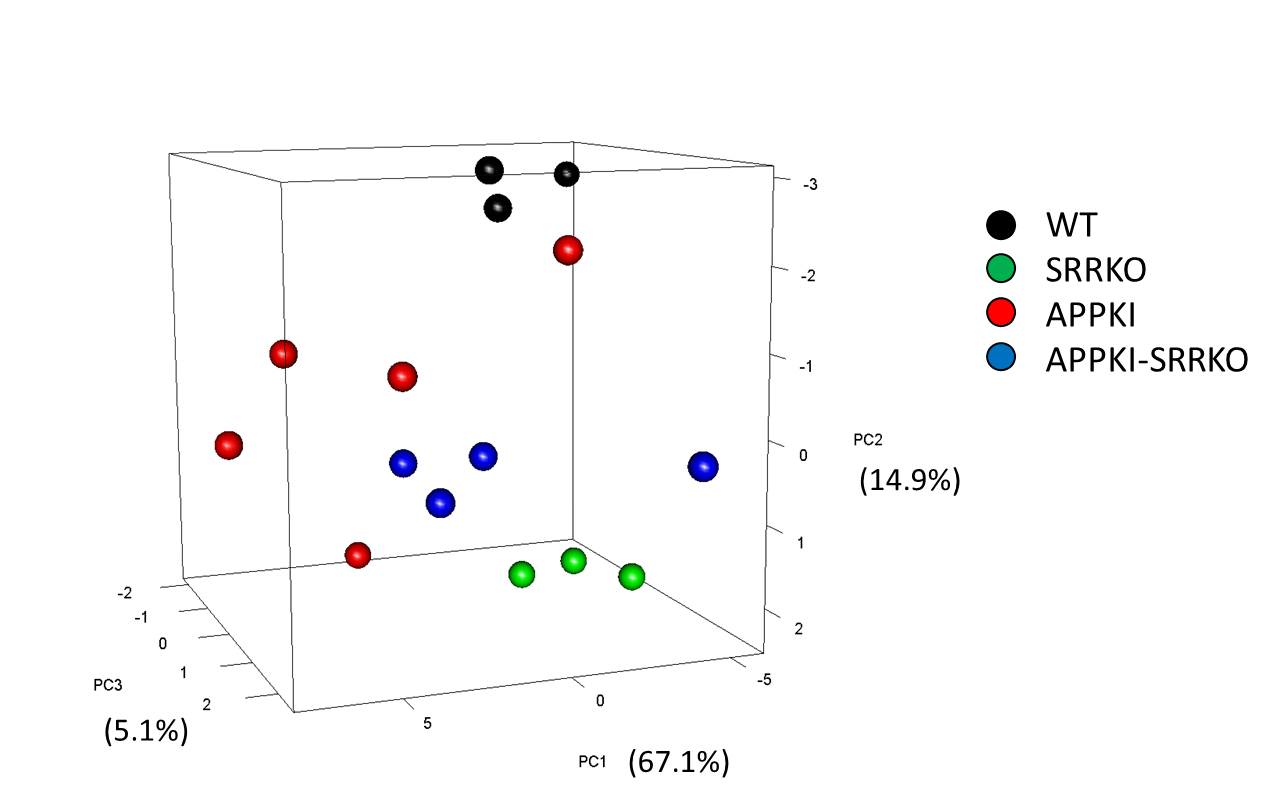


Supplementary Figure 6. Three-dimensional principal component analysis (PCA)

Three-dimensional PCA plots of amino acid concentrations from individual cortical samples. Data of amino acid levels from each mouse were condensed into one single dot in a three-dimensional space that represents the amino acid profile of that mouse. Black plots: WT mice, green plots: SRRKO mice, red plots: APPKI mice, blue plots: APPKI-SRRKO mice. PC1, PC2, and PC3 capture 67.1%, 14.9% and 5.1% of the variance, respectively.

Supplementary Table 1. Antibodies and dilution ratios used for immunofluorescent staining

| Antibodies | Dilution ratios | # Manufacturers or reference |
| --- | --- | --- |
| Rabbit anti-SRR | 1:500 | Inoue et al., 2014 |
| Mouse anti-Aβ | 1:200 | Sigma-Aldrich, cat. No. A1349, clone 4G8 |
| Mouse anti-GFAP | 1:200 | Sigma-Aldrich, cat. no. G3893, clone G-A-5 |
| Mouse anti-NeuN | 1:200 | EMD Millipore, cat. no. MAB 377, Clone A60 |
| Donkey anti-rabbit IgG (Alexa Fluro 488) | 1:500 | Invitrogen, cat no. A21206 |
| Donkey anti-mouse IgG (Alexa Fluro 647) | 1:500 | Invitrogen, cat no. A31571 |
